# Quantifying individual variation in reaction norms: Mind the residual

**DOI:** 10.1111/jeb.13571

**Published:** 2019-12-09

**Authors:** Jip J. C. Ramakers, Marcel E. Visser, Phillip Gienapp

**Affiliations:** ^1^ Department of Animal Ecology Netherlands Institute of Ecology (NIOO‐KNAW) Wageningen the Netherlands; ^2^ Department of Biometris Wageningen University & Research Wageningen the Netherlands; ^3^ Michael‐Otto‐Institut im NABU Bergenhusen Germany

**Keywords:** heteroscedasticity, mixed models, phenotypic plasticity, random regression, random slope

## Abstract

Phenotypic plasticity is a central topic in ecology and evolution. Individuals may differ in the degree of plasticity (individual‐by‐environment interaction (I × E)), which has implications for the capacity of populations to respond to selection. Random regression models (RRMs) are a popular tool to study I × E in behavioural or life‐history traits, yet evidence for I × E is mixed, differing between species, populations, and even between studies on the same population. One important source of discrepancies between studies is the treatment of heterogeneity in residual variance (heteroscedasticity). To date, there seems to be no collective awareness among ecologists of its influence on the estimation of I × E or a consensus on how to best model it. We performed RRMs with differing residual variance structures on simulated data with varying degrees of heteroscedasticity and plasticity, sample size and environmental variability to test how RRMs would perform under each scenario. The residual structure in the RRMs affected the precision of estimates of simulated I × E as well as statistical power, with substantial lack of precision and high false‐positive rates when sample size, environmental variability and plasticity were small. We show that model comparison using information criteria can be used to choose among residual structures and reinforce this point by analysis of real data of two study populations of great tits (*Parus major*). We provide guidelines that can be used by biologists studying I × E that, ultimately, should lead to a reduction in bias in the literature concerning the statistical evidence and the reported magnitude of variation in plasticity.

## Graphical abstract

Mind the residual: unrecognized heteroscedasticity in wild populations may lead to biased estimates of Individual‐by‐Environment interactions (IxE).

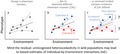

## 1 INTRODUCTION

Behavioural and evolutionary ecologists have long been interested in studying within‐individual variation in animal behaviour and life history (Dingemanse, Kazem, Réale, & Wright, 2010; Piersma & Drent, 2003). For example, the amount of parental care may be altered by offspring needs and explorative behaviour may depend on the time of day (Dingemanse et al., 2010). Similarly, life‐history decisions such as clutch or litter size and timing of reproduction are responsive to the environment, for example food availability or local temperatures (Both, Tinbergen, & Visser, 2000; Brommer, Rattiste, & Wilson, 2008; Réale, McAdam, Boutin, & Berteaux, 2003). Many labile traits are thus phenotypically plastic (Pigliucci, 2001), and this plasticity can be described by reaction norms (Woltereck, 1909). Often these reaction norms are assumed linear, described by an intercept or elevation (phenotype in the average environment, if the environmental average is zero) and a slope (sensitivity of the trait to the environment) (but see Morrissey & Liefting, 2016). Animals may differ from their conspecifics in their mean trait value (Dall, Bell, Bolnick, & Ratnieks, 2012; Réale & Dingemanse, 2010), but also in their degree of phenotypic plasticity (individual‐by‐environment interactions or I × E), leading to changing phenotypic variances across the environmental gradient (Nussey, Wilson, & Brommer, 2007). When these variances have a genetic basis (G × E), this may impact on how populations can respond evolutionarily to environmental change (Merilä, Sheldon, & Kruuk, 2001; Wood & Brodie, 2016; but see Ramakers, Culina, Visser, & Gienapp, 2018). It is hence important to study variation in reaction norms to understand ecological and evolutionary processes in wild populations (Dingemanse et al., 2010; Piersma & Drent, 2003).

Mixed‐modelling approaches are powerful tools to study individual (or genetic) sources of phenotypic variation in natural populations (Nussey et al., 2007; Bolker et al., 2009; Van de Pol & Wright, 2009; Wilson et al., 2010; Dingemanse & Dochtermann, 2013). Random regression models (RRMs) are a special case of mixed‐effects models that allow individuals to differ in their reaction norm elevation as well as slope (Dingemanse & Dochtermann, 2013; Nussey et al., 2007). RRMs can be extended to include an additive genetic effect (e.g. via a pedigree; Henderson, 1988; Kruuk, 2004) in a so‐called “random regression animal model” (RRAM), allowing one to partition I × E into a permanent‐environment (PE × E) and an additive genetic (G × E) component. These methods have been widely used in the evolutionary literature to study the evolutionary potential of a variety of behavioural and life‐history traits (see Gienapp and Brommer (2014) and Appendix S1 in Van de Pol (2012) for relevant overviews).

There are several issues that can lead to misleading conclusions when modelling variation in plasticity (here for simplicity referring to I × E, as opposed to PE × E or G × E), including (a) a lack of power attributable to sampling design and sample size (Martin, Nussey, Wilson, & Réale, 2011; Van de Pol, 2012), (b) using an inappropriate environmental covariate (the “cue” affecting the phenotype) (Gienapp, 2018), and (c) mistaking environmental trends in residual variance (heteroscedasticity) for I × E (see examples below). Here, we focus on the latter. We refer to residual variance as the amount of within‐individual phenotypic variance left unexplained by the statistical model. Although it has been argued to contain biologically relevant information (Cleasby & Nakagawa, 2011; Nicolaus, Brommer, Ubels, Tinbergen, & Dingemanse, 2013; Westneat, Wright, & Dingemanse, 2015), it may cause erroneous inferences of I × E if not appropriately modelled. Nicolaus et al. (2013) found that out of 26 studies of I × E in behavioural and life‐history traits, only 5 allowed for heterogeneity in the residual variances and concluded for their own study (great tit (*Parus major*) clutch size in response to population density) that a RRM with heterogeneous residual variances outperformed a model with homogeneous residual variance. Similarly, Ljungström, Wapstra, and Olsson (2015) found that estimated I × E in egg‐laying date in response to temperature in sand lizards (*Lacerta agilis*) disappeared when residuals were allowed to vary with the environment. Although sample size in this study might have played a role in the apparent lack of I × E, these authors fitted a residual variance for each environment (year), which may have led to severe overfitting of the model. In contrast, Husby et al. (2010) let residual variances only differ between three decadal groups in a RRM estimating I × E in egg‐laying date in great tits. The rationale was that because phenotypic variance increased with temperature, and temperature increased over time due to climate change, fitting decade‐specific residual variances would capture the heteroscedasticity in the RRM, an assumption later found to be false (Ramakers, Gienapp, & Visser, 2018).

The “problem” of heteroscedasticity has long been recognized outside ecology and evolution, for example in the field of animal breeding (Hill, 1984). Although the biological importance of the residual variance is increasingly appreciated in the field of ecology and evolution (Nicolaus et al., 2013; Westneat et al., 2015), there appears to be no clear awareness among evolutionary ecologists about how heteroscedasticity may affect estimates of variation in plasticity (I × E) and how it should be dealt with within the context of RRMs (but see Cleasby & Nakagawa, 2011 for an application outside RRMs). If one is interested in the evolutionary potential of the reaction norm in wild populations (Gienapp & Brommer, 2014; Ramakers et al., 2018), the main goal is usually to get unbiased estimates of I × E and G × E. To achieve this, behavioural and evolutionary ecologists can make use of advocated mixed‐modelling tools (Dingemanse & Dochtermann, 2013; Nussey et al., 2007) and use RRMs in such a way that they effectively account for heterogeneity in residual variances.

In this study, we used a simulation approach to investigate how estimates of I × E, and the statistical power to detect it, are affected by heterogeneity in residual variance not appropriately accounted for in the RRM. We aimed to illustrate in which contexts (the amount of variation in plasticity (I × E), the strength of the environmental dependency of residual variance, the number of individuals and environments, and environmental variability) heteroscedasticity is likely to be problematic in the estimation of I × E and how different residual structures in the RRM deal with this heteroscedasticity. Next, we tested how model selection criteria performed in choosing the model that best fit the simulated data (e.g. with respect to I × E and residual structure). Previous simulation studies have demonstrated how sampling design and size (Martin et al., 2011; Van de Pol, 2012) and the choice of the environmental covariate (Gienapp, 2018) affect the statistical power and predictive accuracy in detecting I × E, so we did not fully explore these aspects here. Finally, we tested how the methodology applied in the simulations performs in the analysis of phenology in two contrasting study populations of great tits. We use the results of our simulations and empirical analysis to extend existing guidelines for students of behavioural and life‐history phenotypic plasticity using random regression models by shifting the focus on heterogeneity in residual variances.

## 2 MATERIALS AND METHODS

### 2.1 Random regression models

A univariate mixed‐model describing the relationship between trait *z* and environment *x* can be written as.

(1) $$z_{\mathrm{ij}}=a_{0}+a_{i}+bx_{\mathrm{ij}}+e_{\mathrm{ij}},$$

where $z_{\mathrm{ij}}$ is the *j* ^th^ phenotype of the *i* ^th^ individual, and the linear function of $z_{\mathrm{ij}}$ on environment $x_{\mathrm{ij}}$ is characterized by the population‐mean intercept $a_{0}$ plus the individual deviation $a_{i}∼N\left(0,\sigma_{a}^{2}\right)$, the population‐mean slope *b* and the error term $e_{\mathrm{ij}}∼N\left(0,\sigma_{e}^{2}\right)$. This so‐called random‐intercept model (RIM) can be extended to a random regression model (RRM), where each individual is allowed to not only have a different intercept, but also a different slope $b_{i}$:

(2a) $$z_{\mathrm{ij}}=a_{0}+a_{i}+\left(b+b_{i}\right)x_{\mathrm{ij}}+e_{\mathrm{ij}},$$

where

$${\left[\begin{array}{c} a\\ b \end{array}\right]}_{i}∼N\left(\left[\begin{array}{c} 0\\ 0 \end{array}\right],{\left[\begin{array}{cc} \sigma_{a}^{2} & \sigma_{a,b}\\ \sigma_{a,b} & \sigma_{b}^{2} \end{array}\right]}_{i}\right).$$

The error term in Equation (2a) can be assumed to come from a univariate normal distribution as above, but may sometimes itself be described by some function of the environment and is modelled as.

(2b) $$z_{\mathrm{ijk}}=a_{0}+a_{i}+\left(b+b_{i}\right)x_{\mathrm{ijk}}+e_{\mathrm{ijk}},$$

where *k* denotes a group categorizing similar environments (e.g. groups of years with low, intermediate and high temperatures), and where

$$e_{\mathrm{ijk}}∼N\left({\left[\begin{array}{c} 0\\ \vdots \\ 0 \end{array}\right]}_{\mathrm{ij}},{\left[\begin{array}{ccc} \sigma_{e,1}^{2} & \cdots & 0\\ \vdots & \ddots & \vdots \\ 0 & \cdots & \sigma_{e,k}^{2} \end{array}\right]}_{\mathrm{ij}}\right).$$

Note that in reality, error variance ($\sigma_{e}^{2}$) is more likely to vary with *x* in a more continuous and gradual fashion (whether linearly or not). When $\sigma_{e}^{2}$ varies with *x* in a directional fashion (e.g. a linear increase or decrease), the model of Equation (2a) will likely fail to estimate variation in reaction norm slopes ($\sigma_{b}^{2}$) accurately (i.e. the estimate may be inflated because the RRM may “force” reaction norms to converge at one end of the range of *x* and diverge at the other). Model (2b) should in this case be more appropriate. In empirical datasets, however, we can measure the association between phenotypic variation ($\sigma_{z}^{2}$) and the covariate of interest (*x*) but it will be unclear whether this association is attributable to heterogeneity in $\sigma_{e}^{2}$, $\sigma_{b}^{2}$ or both.

### 2.2 Simulation 1: effect of residual variance structure on estimates and detection rates of I × E

We tested with simulated data how the estimation of variance in reaction norm slopes, as well as the statistical power to detect it, differed between models with a homogeneous and heterogeneous residual structure. Specifically, we tested how this difference was mediated by the following factors (see Table 1): (a) the mean number of observations per individual (*N_o_*), (b) the total number of different environments (*N_x_*), (c) the variability in the environment ($\sigma_{x}^{2}$), (d) the variation in slopes ($\sigma_{b}^{2}$) and (e) an association between phenotypic variance and the environment caused by a (linear) correlation between residual variance and the environment ($r_{\sigma_{e}^{2},x}$). Every combination of parameters (Table 1) was simulated 1,000 times.

**Table 1:** Parameter input in the simulation testing the effect of the residual variance structure in the RRMs to detect variation in reaction norm slopes

Parameter	Description	Tested values
1. *N_o_*	Number of observations per individual	2, 5
2. *N_x_*	Number of different environments (years)	20, 40
3. $\sigma_{x}^{2}$	Variance in the environment	1, 2, 3
4. $\sigma_{b}^{2}$	Variance in reaction norm slopes	0.003, 0.3, 1.0
5. $r_{\sigma_{e}^{2},x}$	Coefficient of correlation between residual variance ($\sigma_{e}^{2}$) and the environment (*x*).	0.01, 0.2, 0.5, 0.8

Environments were randomly drawn from a normal distribution, $x_{j}∼N\left(0,\sigma_{x}^{2}\right)$. Residual variance $(\sigma_{e}^{2}$) was assumed to be a linear function of the environment. We drew values for $\sigma_{e}^{2}$ in each environment (mean = 10) according to $r_{\sigma_{e}^{2},x}$ such that

$$\sigma_{e,j}^{2}=\left[r_{\sigma_{e}^{2},x}\right]\left[\sigma_{res_{x,q}}\right]x_{j}+{\left[res_{x∼q}\right]}_{j}+\sigma_{x}\sqrt{1-{\left[r_{\sigma_{e}^{2},x}\right]}^{2}}+10,$$

where *r* is the correlation coefficient ($r_{\sigma_{e}^{2},x})$ and ${\left[res_{x∼q}\right]}_{j}$ is the residual of the linear regression between $x_{j}$ and a preliminary variable $q_{j}∼N\left(0,1.5\right)$. The procedure was repeated as often as necessary to reach 2.8 < *var*($\sigma_{e}^{2}$) < 3.2, to ensure that the effects of $r_{\sigma_{e}^{2},x}$ and *var*($\sigma_{e}^{2}$) in the RRMs were not confounded. Each individual (*N* = 500) with *N_o_* observations was randomly assigned to a breeding cohort within the range of *x*. Individuals randomly received a value for the intercept (*a_i_*) and slope (*b_i_*) (population mean = 0) and their phenotypes in environment $x_{j}$ were determined following Equation (2b), with $e_{\mathrm{ij}}$—not $e_{\mathrm{ijk}}$—drawn from the vector of residual variances generated above. We varied $\sigma_{b}^{2}$ (Table 1) but fixed $\sigma_{a}^{2}$ to 3; $\sigma_{a,b}$ was assumed to be zero. The three scenarios for $\sigma_{b}^{2}$ were chosen based on the estimates gained from studies listed in Table 3 in Nicolaus et al. (2013), which we used to derive the slope variance in proportion to the intercept variance. That is, for all studies that fitted a model on data on the original (nonstandardized) scale and reported estimates of ${\hat{\sigma }}_{a}^{2}$ and ${\hat{\sigma }}_{b}^{2}$ (20 pairs of estimates from 6 studies) we divided the ${\hat{\sigma }}_{b}^{2}$ by ${\hat{\sigma }}_{a}^{2}$ and deduced from that 0.001, 0.1 and 0.33 as small, intermediate and large proportions of slope variance in relation to intercept variance. In our simulations, this meant $\sigma_{b}^{2}=0.001\;\sigma_{a}^{2}=0.003$, $\sigma_{b}^{2}=0.1\;\sigma_{a}^{2}=0.3$ or $\sigma_{b}^{2}=0.33\;\sigma_{a}^{2}=1$, respectively (Table 1). We used $\sigma_{b}^{2}=0.003$ as a null scenario (variance close to zero).

With simulated environments and phenotypes in place, we fitted RRMs with five different variance structures, using the package “nlme” (Pinheiro, Bates, DebRoy, Sarkar, & Team, 2017). Model 1 had a homogeneous residual variance (Equation 2a); the residual structures in the next four models were variations of Equation (2b). For Model 2 and 3, environments were categorized into $k=N_{x}/5$ or $k=N_{x}/10$ equal‐interval groups of similar environments, respectively, and estimated residual variance ${\hat{\sigma }}_{e}^{2}$ was partitioned accordingly to capture environmental trends. For Models 4 and 5, again $k=N_{x}/5$ or $k=N_{x}/10$, but this grouping was done based on consecutive environments, rather than similar environments (tantamount to random grouping). Models 4 and 5 served as “controls” to test whether a heterogeneous residual structure per se affects model performance (note that the number of degrees of freedom, that is the difference in the number of parameters, increases by 1 with each additional residual variance component).

From each model we extracted ${\hat{\sigma }}_{b}^{2}$. To test the significance of I × E, we compared each RRM to a RIM (keeping the same residual variance structure) with a likelihood‐ratio test (LRT) with 1 degree of freedom. We extracted the proportion of tests with *p* < .05 from the 1,000 simulation runs (“power”). We used the LRT for hypothesis testing for illustrative purposes, as this is an intuitive and preferred method by many researchers, and since it provides a way to compare the power of our models between scenarios. Note, however, that the LRT can be conservative in practice and may not be the preferred method of testing variance components (e.g. Bolker, 2008; Goldman & Whelan, 2000).

### 2.3 Simulation 2: distinguishing heterogeneous residual variance from I × E

When environmental heterogeneity in phenotypic variance ($\sigma_{z}^{2}$) is present in the data, the question is whether RRMs can be used to disentangle whether this is caused by heterogeneity in $\sigma_{e}^{2}$, $\sigma_{b}^{2}$ (I × E), or both. In the second simulation, we repeated the analysis of above but focused specifically on relative model performance. We fixed *N_o_* to 2 or 5, *N_x_* to 40 and $\sigma_{x}^{2}$ to 2. We simulated six scenarios, that is all combinations of $\sigma_{b}^{2}=0.003\text{or}1$ and $r_{\sigma_{e}^{2},x}=0.01$, 0.2 or 0.8, and assessed relative model performance using Akaike's information criterion (AIC; Burnham & Anderson, 2002). The rationale was that if, for example, heterogeneity in $\sigma_{e}^{2}$ was present but I × E was not, a RRM with a homogeneous residual structure (Equation 2a) may perform better (and have a higher penalized likelihood) than a RIM that incorporated a heterogeneous residual structure. In such a scenario, one would erroneously conclude that I × E was sizeable, whereas in reality it was too small to be detected statistically. Note that the reverse could equally be true.

We fitted Models 1 to 3 as well as their random‐intercept counterparts as described above for Simulation 1. For simplicity, we regarded the best fitting model as the most parsimonious one (i.e. with the fewest degrees of freedom) within 2 AIC units from the model with the lowest AIC value.

### 2.4 Applying RRMs with different residual structures to real data

As a last step, we aimed to illustrate how different treatments of the residual variance in RRMs affected estimates of I* × *E in real data, and how model selection criteria in this context can provide misleading conclusions as to the presence of I × E depending on the residual variance specification. We used individual data of egg‐laying dates in two of our long‐term study populations of the great tit (*P. major*) at the Hoge Veluwe (HV; 52°01'57"N 5°52'05"E; $N_{\text{broods/females}}=4890/3028$) and the island of Vlieland (VL; 53°18′N, 5°03′E; $N_{\text{broods/females}}=5250/3131$; note that excluding birds with only one or two broods did not affect the results (not shown here)). For a full description of the data collection and methods, see Ramakers et al. (2018).

We first defined the “basic” RIM for laying date in our populations in package “lme4” (Bates et al., 2018). The *j* ^th^ laying date of the *i* ^th^ female in the *l* ^th^ nest box and the *h* ^th^ year is described as.

(3) $$z_{ijlh\left(m\right)}=a_{0}+a_{i}+c{\bar{T}}_{i}+b\left(T_{\mathrm{ij}}-{\bar{T}}_{i}\right)+{\text{age}}_{\mathrm{ij}}\left(+{\;\text{village}}_{m}\right)+{\text{nb}}_{l}+{\text{yr}}_{h}+e_{ijlh\left(m\right)},$$

where $a_{0}$ is the population intercept, $a_{i}$ is the individual deviation from the population intercept (i.e. a random effect of female identity), *c* the average slope of the phenotype against the average spring temperature encountered by individual *i* (${\bar{T}}_{i}$) and *b* the average slope for the individual‐centred temperature $\left(T_{\mathrm{ij}}-{\bar{T}}_{i}\right)$, ${\text{age}}_{\mathrm{ij}}$ the female's age (first‐year breeder or older) at the time of breeding, ${\text{village}}_{m}$ (in or outside the village; only at VL, hence the parentheses around index *m*), ${\text{nb}}_{l}$ and ${\text{yr}}_{h}$ the nest box and year, respectively (as random effects) and $e_{ijlh\left(m\right)}$ the residual term. The model of Equation (3) (called Model i) was compared to five different variations on it (Model ii–vi, comparing residual structures and RIMs vs. RRMs; see Table 2).

**Table 2:** Model specifications for great tit laying date (z) in the Hoge Veluwe and Vlieland populations

Model	Equation	*k*
i	$z_{ijlh\;\left(m\right)}=a_{0}+a_{i}+b\left(T_{\mathrm{ij}}-{\bar{T}}_{i}\right)+c{\bar{T}}_{i}+{\text{age}}_{\mathrm{ij}}\left(+{\text{village}}_{m}\right)+{\text{nb}}_{l}+{\text{yr}}_{h}+e_{ijlh\left(m\right)}$	1
ii	$z_{ijklh\;\left(m\right)}=a_{0}+a_{i}+b\left(T_{\mathrm{ij}}-{\bar{T}}_{i}\right)+c{\bar{T}}_{i}+{\text{age}}_{\mathrm{ij}}\left(+{\text{village}}_{m}\right)+{\text{nb}}_{l}+{\text{yr}}_{h}+e_{ijklh\left(m\right)}$	9
iii	$z_{ijklh\;\left(m\right)}=a_{0}+a_{i}+b\left(T_{\mathrm{ij}}-{\bar{T}}_{i}\right)+c{\bar{T}}_{i}+{\text{age}}_{\mathrm{ij}}\left(+{\text{village}}_{m}\right)+{\text{nb}}_{l}+{\text{yr}}_{h}+e_{ijklh\left(m\right)}$	4/5
iv	$z_{ijlh\;\left(m\right)}=a_{0}+a_{i}+\left(b+b_{i}\right)\left(T_{\mathrm{ij}}-{\bar{T}}_{i}\right)+c{\bar{T}}_{i}+{\text{age}}_{\mathrm{ij}}\left(+{\text{village}}_{m}\right)+{\text{nb}}_{l}+{\text{yr}}_{h}+e_{ijlh\left(m\right)}$	1
v	$z_{ijklh\;\left(m\right)}=a_{0}+a_{i}+\left(b+b_{i}\right)\left(T_{\mathrm{ij}}-{\bar{T}}_{i}\right)+c{\bar{T}}_{i}+{\text{age}}_{\mathrm{ij}}\left(+{\text{village}}_{m}\right)+{\text{nb}}_{l}+{\text{yr}}_{h}+e_{ijklh\left(m\right)}$	9
vi	$z_{ijklh\;\left(m\right)}=a_{0}+a_{i}+\left(b+b_{i}\right)\left(T_{\mathrm{ij}}-{\bar{T}}_{i}\right)+c{\bar{T}}_{i}+{\text{age}}_{\mathrm{ij}}\left(+{\text{village}}_{m}\right)+{\text{nb}}_{l}+{\text{yr}}_{h}+e_{ijklh\left(m\right)}$	4/5

Note

*k* is the number of residual variances estimated, obtained by dividing the number of years by N_X_ (homogeneous variance), 5 (resulting in 9 groups) or 10 (resulting in 4 or 5 groups in HV and VL, respectively). See text for explanation for other symbols.

Models were specified in the package “MCMCglmm” (Hadfield, 2010), since the “nlme” and “lme4” packages do not allow for the inclusion of crossed random effects or heterogeneous residual variances, respectively. We used default normal priors for fixed effects, inverse Wishart priors for the residual variance (*V* = diag(*k*) and nu = 0.002) and parameter‐expanded priors for the random effects (*V* = diag(*d*), nu = *d*, alpha.mu = 0, alpha.V = diag(*d*)*625, where *d* is the matrix dimension). The parameter‐expanded priors are preferred for variance components (but are not implemented in the residual structure) because of their superior mixing properties, especially when empirical values lie close to zero (see discussion in Hadfield, 2018). Models were run for a total of $10.1\cdot 10^{6}$ MCMC steps, with a burn‐in period of 10^5^ samples and a thinning interval of 10^4^. We report the posterior estimates of slope variance from Models iv–vi (Table 2) as well as the differences in deviance information criteria (ΔDIC) between models as a measure of relative model performance (Spiegelhalter, Best, Carlin, & Linde, 2002). Since issues have been raised about using DIC for model comparison in certain contexts (Hadfield, 2018; Spiegelhalter et al., 2002), we used a conservative but reasonable cut‐off point of 6 DIC units from the most parsimonious model, analogous to ΔAIC=6 recommended for AIC (Richards, 2005; Burnham, Anderson, & Huyvaert, 2011; see also Spiegelhalter et al., 2002).

## 3 RESULTS

### 3.1 Effect of residual variance structure on estimates and detection rates of I × E

**Figure 1:**
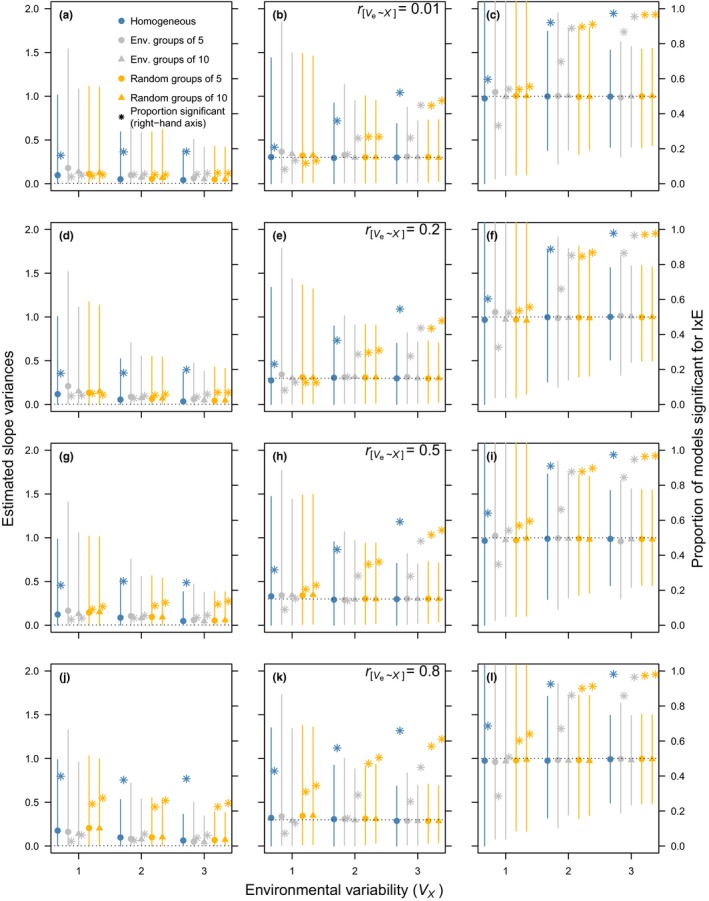
Estimated slope variances (median + 95% CI; left‐hand axis) and proportion of significant (*p* < .05) models (“power”; asterisks, right‐hand axis) from different random regression analyses on different simulated scenarios (*N_o_* = 2 and *N_x_* = 20 in all panels; see Table 1). From top to bottom: change in $r_{\sigma_{e}^{2},x}$; from left to right: decrease in simulated $\sigma_{b}^{2}$ increases (0.003, 0.3, 1.0), denoted with horizontal dotted lines. The horizontal axis displays the environmental variability ($\sigma_{x}^{2}$); different colours and symbols display the estimates from models with different residual structures (blue: homogeneous residual structure; grey and yellow: heterogeneous residual structure based on similar environments and through random grouping, respectively, using groups of 5 (circles) or 10 (triangles) environments)

**Figure 2:**
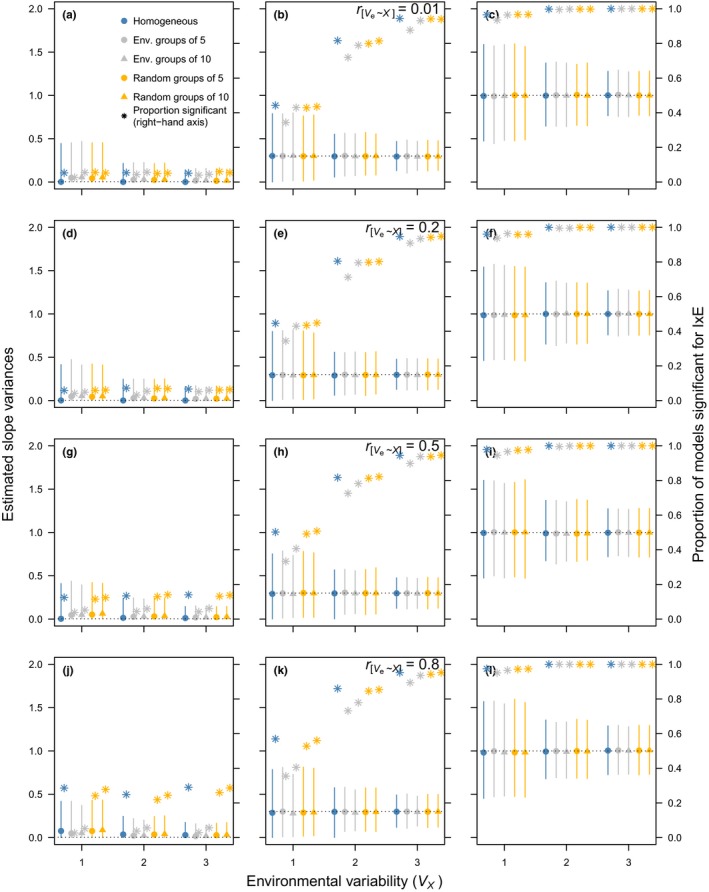
Estimated slope variances (median + 95% CI; left‐hand axis) and statistical power (right‐hand axis) from different random regression models on different simulated scenarios (*N_o_* = 5 and *N_x_* = 20 in all panels; see Table 1). See Figure 1 for a description of each panel and the different symbols

Data structure and sample size mediated the effect of the residual variance structure on both the estimates of I × E ($\sigma_{b}^{2}$) and the probability of (falsely) detecting it using likelihood‐ratio tests. For brevity, we describe here only the scenarios where *N_o_* = 2, *N_x_* = 20 (Figure 1) and *N_o_* = 5, *N_x_* = 20 (Figure 2; see Figures S1 and S2 for scenarios where *N_x_* = 40). When $\sigma_{b}^{2}=0.003$, RRMs consistently overestimate $\sigma_{b}^{2}$, regardless of the RRM structure deployed (Figure 1a,d,g,j); this bias decreases across contexts as the environment becomes more variable ($\sigma_{x}^{2}$; horizontal axes). As $r_{\sigma_{e}^{2},x}$ increases (Figure 1, top to bottom), fitting a heterogeneous residual variance structure based on grouped environments slightly reduces the bias in the estimates when the number of groups is low (two groups of ten environments); that is, the median values move closer to the input value. Fitting more variances (four groups of five environments) in fact increases the imprecision of the estimates. When $\sigma_{b}^{2}=0.3$, the bias in estimates is less pronounced, but again fitting “too many” residual variances increases the imprecision (Figure 1b,e,h,k). When $\sigma_{b}^{2}=1$ (Figure 1c,f,i,l), median slope estimates almost invariably match the input values reasonably well, regardless of levels of heteroscedasticity and the fitted model, but precision improves substantially as $\sigma_{x}^{2}$ increases. Thus, the precision of I × E estimates greatly depends on the variability in the environment and when real $\sigma_{b}^{2}$ is small, failure to fit the proper residual structure may lead to imprecise estimates of I × E (Figure 1). An increase in the number of observations per individual can remedy these issues substantially (Figure 2), as can, to a lesser extent, an increase in the number of environments (Figures S1 and S2).

Fitting a heterogeneous residual variance structure based on similar environments systematically leads to a reduction in the power (*P*) to detect I × E when $\sigma_{b}^{2}=0.003$ (*P « *0.1; Figures 1 and 2, secondary vertical axis). We would therefore (rightfully) accept the null hypothesis that I × E was absent. Conversely, fitting homogeneous residual variance, or “random” heterogeneous residual variance, increases *P* as $r_{\sigma_{e}^{2},x}$ increases, leading to the erroneous conclusion that I × E ≫ 0. When $\sigma_{b}^{2}=1$, *p* > .8 in highly variable environments (Figure 1c,f,i,l) and as the number of observations per individual increases, the influence of $\sigma_{b}^{2}$ is further reduced (Figure 2c,f,i,l). An exception is when the residual variance is partitioned into environmental blocks of 5: even at $\sigma_{b}^{2}=1$, when $N_{o}=2$ (Figure 1), “power” to detect slope variance typically falls below 0.8 at moderate environmental variability ($\sigma_{x}^{2}=2$) when the residual variance is partitioned too excessively. Again, this issue disappears when we have more observations per individual (Figure 2), but at $\sigma_{b}^{2}=0.003$ the inappropriate residual structures keep the false‐positive rate unacceptably high (P≫0.2). Thus, when true $\sigma_{b}^{2}$ is small and $r_{\sigma_{e}^{2},x}$ is strong, fitting the right (heterogeneous) residual structure is crucial to correctly infer statistical evidence for I × E. Moreover, increasing the precision in estimates of I × E and statistical power to detect it when it is there is achieved more easily by increasing *N_o_* than by increasing *N_x_* (Figures S1 and S2).

### 3.2 Distinguishing heterogeneous residual variance from I × E

**Figure 3:**
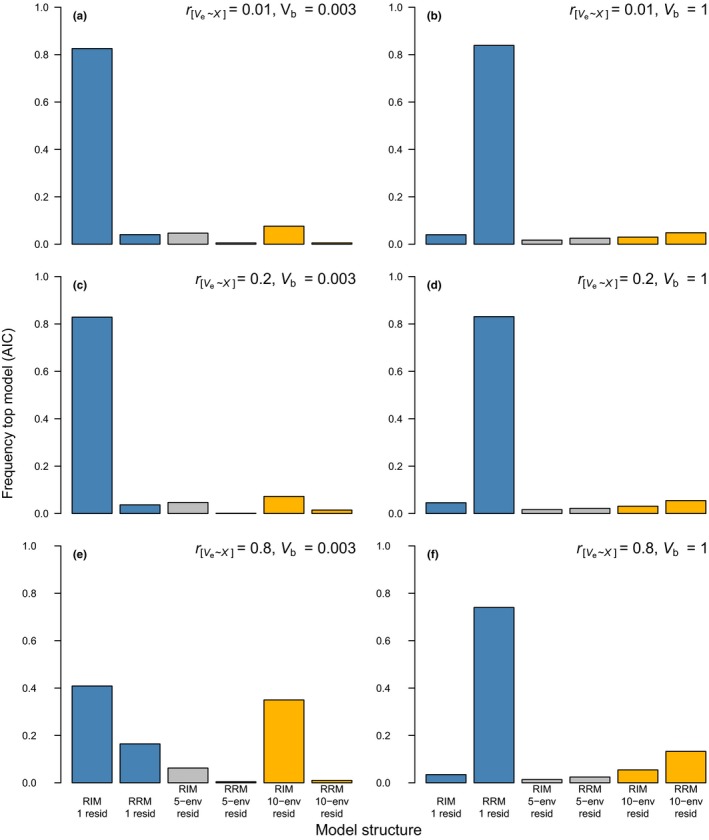
Frequency with which each model is chosen as the top model (based on ΔAIC < 2 and parsimony) under different scenarios (all *N_x_* = 40, *N_o_* = 2 and $\sigma_{x}^{2}$ = 2). Top to bottom: increased heterogeneity in residual variance ($r_{\sigma_{e}^{2},x}$); left to right: increased slope variance ($\sigma_{b}^{2}$). Fitted models (horizontal axes) were random‐intercept models (RIM) or random regression models (RRM) with a homogeneous residual variance structure (“1 resid”; blue bars), heterogeneous partitioned into groups of 5 (“5‐env”; grey bars) or groups of 10 environments (“10‐env”; orange bars). Note that the meaning of the colours in this figure differs from that in Figures 1 and 2

**Figure 4:**
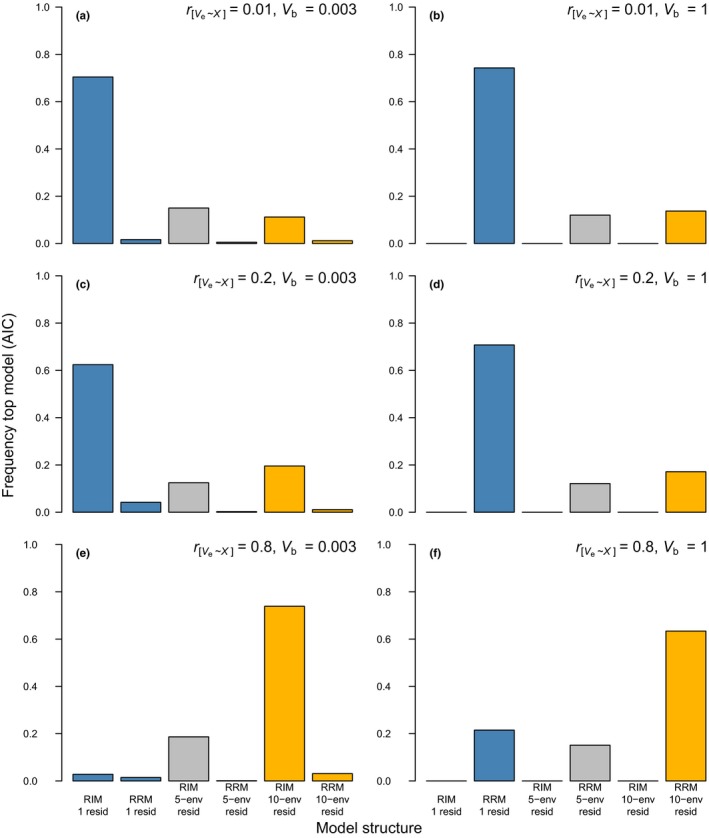
Frequency with which each model is chosen as the top model (ΔAIC < 2) under different scenarios (all *N_x_* = 40, *N_o_* = 5 and $\sigma_{x}^{2}$ = 2). See the caption to Figure 3 for an explanation of the different scenarios and the description of the different colours

Whenever there is an association between $\sigma_{z}^{2}$ and the environment *x*, simple model comparison using AIC is effective at arriving at the qualitative conclusion of whether or not there is statistical evidence for I × E. That is, a combined proportion > 0.8 of models that appeared as the best model in the selection processes were either random regression models (RRMs) when simulated $\sigma_{b}^{2}=1$ or random‐intercept models (RIMs) when simulated $\sigma_{b}^{2}=0.003$ (see Figure 3 for *N_o_* = 2 and Figure 4 for *N_o_* = 5). However, with few observations per individual (Figure 3), selection of the “correct” residual variance structure—matching the simulated data (i.e. homogeneous vs. heterogeneous)—was achieved at a rate *«* 0.8. For example, with a moderate heterogeneity in residual variance ($r_{\sigma_{e}^{2},x}=0.2$), models with a homogeneous residual structure were chosen most often (Figure 3c,d). When $\sigma_{e}^{2}=0.003$ and $r_{\sigma_{e}^{2},x}=0.8$ (Figure 3e,f), both models with and without a heterogeneous residual structure (with 10‐env. groups) were selected at competing rates.

As expected, increasing *N_o_* improved model selection (Figure 4). At $r_{\sigma_{e}^{2},x}=0.2$, the proportion of selected models having a homogeneous residual variance decreased at *N_o_* = 5 compared to *N_o_* = 2 (note the rise in the orange and grey bars in Figure 4c,d). At $r_{\sigma_{e}^{2},x}=0.8$, the vast majority of selected models was again correctly defined as either RIM or RRMs, and additionally had a heterogeneous residual structure (0.93 and 0.79, respectively; Figure 4e,f).

### 3.3 Modelling I × E in great tit egg‐laying dates

The HV and VL great tit populations differ in the degree of plasticity in egg‐laying date with respect to spring temperature (Table 3). At HV, the best model arising from DIC model selection was the random‐intercept model with a heterogeneous residual structure (Model ii in Tables 2 and 3). In this population, raw annual phenotypic variance in laying dates correlates positively with mean spring temperature (coefficient + bootstrapped 95% CI: 2.39 [0.702, 4.502]). As the estimate and 95% HPDI for ${\hat{\sigma }}_{b}^{2}$ in Model v show, I × E is limited in this population, so the association between $\sigma_{z}^{2}$ and temperature is not caused by individually differing reaction norms but to other, unmeasured (residual) factors. Comparing RIMs and RRMs while fitting a homogeneous residual structure (Model i vs. iv), this conclusion changes radically: now the DIC values suggest a strong preference for Model iv over Model i (ΔDIC = 41.8) with ${\hat{\sigma }}_{b}^{2}$ 4.4 to 4.9 times the size of that of Model v or vi.

**Table 3:** Results of the RRMs on great tit laying dates from the Hoge Veluwe and Vlieland populations

Model	Random effects	Structure $\sigma_{e}^{2}$	Envs. grouped by	No. of residual groups	ΔDIC	${\hat{\sigma }}_{b}^{2}$ (95% HPDI)
*Hoge Veluwe*						
i	Y + NB +I	Ho	44 (*N_x_*)	1	159.0	‐
**ii**	**Y + NB +I**	**He**	**5**	**9**	**2.3**	‐
iii	Y + NB +I	He	10	4	88.4	‐
iv	Y + NB +IxE	Ho	44	1	117.1	0.168 (0.018, 0.336)
v	Y + NB +IxE	He	5	9	0	0.034 (0.000, 0.123)
vi	Y + NB +IxE	He	10	4	85.5	0.039 (0.000, 0.135)
*Vlieland*						
i	Y + NB +I	Ho	47 (*N_x_*)	1	867.4	‐
ii	Y + NB +I	He	5	9	230	‐
iii	Y + NB +I	He	10	5	392.3	‐
**iv**	**Y + NB +IxE**	**Ho**	**47**	**1**	**0**	**1.893 (1.428, 2.322)**
v	Y + NB +IxE	He	5	9	19.8	0.963 (0.428, 1.545)
vi	Y + NB +IxE	He	10	5	39.4	1.511 (1.032, 2.068)

Note

Y = year, NB = nest box, I = individual, I × E = individual‐by‐environment interaction, Ho = homogeneous residual variance, He = heterogeneous residual variance, *N_x_* = number of environments (here: years). The best models (based on DIC and parsimony) are marked in bold.

At VL, the best supported model is a RRM with a homogeneous residual structure (Model iv in Tables 2 and 3). In this population, there is clear evidence for individual reaction norms differing in temperature sensitivity and this evidence is picked up by the RRMs regardless of its residual structure (see ${\hat{\sigma }}_{b}^{2}$ and 95% HPDIs for Models iv–vi), concurring with our simulation results (see Figures 1 and 2). Importantly, however, the effect size critically depends on the residual structure. Unlike the HV population, raw phenotypic variances in laying date at VL do not correlate with temperature (0.961 [–1.258, 3.562]). The lack of this association suggests that $\sigma_{z}^{2}$ covaries nonlinearly with temperature and that this is due to crossing reaction norms and not due to heterogeneity in residual variance (see Figure S3).

## 4 DISCUSSION

We have shown with simulations that the precision with which I × E can be estimated depends on the level of heterogeneity in residual variance in the data and the way this heterogeneity is subsequently modelled. Importantly, substantial variability in the environment is a prerequisite for reliably estimating—and detecting—variance in reaction norm slopes, although this effect wanes when individuals have observations in many (>2) environments (cf. Van de Pol, 2012). When these conditions are not met, failure to model heteroscedasticity in residuals adequately may strongly impair precision of estimates and the ability of statistical tests to correctly reject or maintain the null hypothesis. In our empirical example, the effect of the modelled residual structure on the magnitude of estimated I × E (bias) was even more pronounced. We would therefore encourage due caution before proceeding to estimate I × E in observational studies (cf. Nicolaus et al., 2013).

Several studies have alluded to both the biological and statistical importance of heteroscedasticity (e.g. Cleasby & Nakagawa, 2011; Nicolaus et al., 2013; Westneat et al., 2015). However, in the oft‐cited mixed‐model “how‐to’”paper by Dingemanse and Dochtermann (2013), the implications of heteroscedasticity on model performance and the correct application of alternative methods are not discussed. The same is true for Nussey et al.’s (2007) guideline paper for the use of random regression models in studies of phenotypic plasticity. Previous simulation studies on the subject of random regression models (Gienapp, 2018; Martin et al., 2011; Van de Pol, 2012) simulated data under the assumption of constant residual variance. Our study adds to previous work by studying heteroscedasticity in a random regression framework with simulated (and empirical) data with the specific aim to illustrate its effect on model estimates and inference from hypothesis testing.

Cleasby and Nakagawa (2011) perhaps give the most complete practical guidance for ecologists on how to identify and correctly model heteroscedasticity in a standard linear‐model framework. They suggested (1) using heteroscedasticity‐consistent standard error estimations or (2) fitting a generalized least‐squares model. In their example analysis on experimental data (tarsus length as a function of feeding treatment and sex in house sparrows *Passer domesticus*), the latter was achieved by fitting a residual variance for each treatment–sex combination. In our RRMs, the covariate (the environment) was continuous and grouping therefore had to be done “experimentally” by varying the groups and selecting the most plausible model. Nicolaus et al. (2013) did this by comparing two heterogeneous residual structures when testing variation in plasticity of clutch size with respect to population density and found that partitioning residual variance by year—as opposed to two groups of environments—yielded the most plausible model. Our simulation results suggest that fitting a heterogeneous residual structure with many groups will be problematic when sample sizes are small (see Figure 1), potentially due to overfitting. This may also have been the case, for example in a study on egg‐laying dates in sand lizards, in which the residual variance in the RRM was estimated for each year (Ljungström et al., 2015). Fitting a homogeneous residual variance in that study yielded ${\hat{\sigma }}_{b}^{2}=10.4\left(\pm 3.4\;\text{SE}\right)$, whereas it decreased to 0 when fitting heterogeneous residual variance. Although the log‐likelihood of the model improved considerably, the best model may actually have been a compromise between the two. Fitting too few groups, on the other hand, may not adequately deal with heteroscedasticity and lead to overestimation of ${\hat{\sigma }}_{b}^{2}$. We did not explore “annual” residual variances in our simulations because the models could not be fit under certain conditions. We therefore strongly suggest that a “sensitivity analysis” be conducted by changing the number of residual variances stepwise and judge relative model performance using information criteria. Caution is, however, always warranted when the sample size is low, and it may be reasonable to assume that fitting a residual variance for each environment will result in severe overfitting and potentially erroneous conclusions. Ideally, when $\sigma_{e}^{2}$ changes in a continuous fashion, it should be modelled as such; a model allowing this would be a parsimonious alternative to fitting separate residual variances (Equation 2b). Although this model can be fitted using the “nlme” package, we did not include it in the simulations since, to our knowledge, it is not a practical solution for many of the frequently used software packages.

Fitting residual variance for different groups of environments is an effective way of dealing with heteroscedasticity, but obtaining reliable estimates of I × E naturally starts with the identification of the best “null” model describing the trait of interest, including the fixed effects on which the variance components are conditioned. Typical reproductive traits such as egg‐laying date and clutch size, for example, vary with age. If the phenotypic response to the environment changes with age (A × E; e.g. Van de Pol, Osmond, & Cockburn, 2012), individual variation in reaction norm slopes may in fact reflect (unobserved) A × E and not I × E (see discussion in Van de Pol, 2012); failing to fit the appropriate age structure in the model may lead to heteroscedasticity and, in turn, to the erroneous conclusion of I × E. Cleasby and Nakagawa (2011) give a comprehensive account of ecological factors generating changes in residual variances across environmental gradients. Their main point, and that of others (e.g. Westneat et al., 2015), is that heteroscedasticity is a perfectly natural biological component of the data that, rather than being just statistical “nuisance” (Erceg‐Hurn & Mirosevich, 2008), should inspire researchers to formulate new hypotheses and build their models accordingly.

### Recommendations for evolutionary and behavioural ecologists

The results of our simulations and empirical data analysis can be used to draw up a set of guidelines for behavioural and evolutionary ecologists interested in phenotypic plasticity. Important recommendations involving RRMs, and heteroscedasticity more generally, have been made by others (Nussey et al., 2007; e.g. Cleasby & Nakagawa, 2011; Martin et al., 2011; Van de Pol, 2012; Dingemanse & Dochtermann, 2013; Nicolaus et al., 2013; Gienapp, 2018). Note, also, that random regression techniques were originally developed mainly for the field of animal breeding (Henderson, 1982; Schaeffer, 2004) and developments of tools mainly take place within this field. There are sophisticated statistical tools available for modelling heteroscedasticity (see Lee & Nelder, 2006; Rönnegård, Felleki, Fikse, Mulder, & Strandberg, 2010) that may be preferred in some contexts on biological and/or statistical grounds. We, however, would like to present guidelines that can be used within the R environment in software packages and methods that many ecologists will be familiar with (e.g. “nlme” (Pinheiro et al., 2017), “MCMCglmm” (Hadfield, 2010) and “ASReml‐R” (Butler, Cullis, Gilmour, & Gogel, 2009; Gilmour, Gogel, Cullis, & Thompson, 2009)).

When it comes to random regression models to estimate I × E (and/or G × E), we suggest the following steps (but particularly step 1, 2 and 4) be given sufficient thought:

1. *Plot raw phenotypic variance against the environmental covariate*. Plotting the data prior to analysis can sometimes be quite revealing, since it may give us an idea of whether and how we can expect variances to change with the environment. This may be helpful in deciding by how many groups residual variance in the RRM may need to be modelled. Furthermore, as a reality check, we can compare the plot to a plot of individual reaction norms drawn from RRMs (using “best linear unbiased predictors” (BLUPs) or their equivalents) and visually check if the trends in phenotypic variation match the estimated individual reaction norms.
2. *Compare RRMs with several different residual structures using information criteria.* To our knowledge, there is no clear guideline as to how many residual variances are reasonable, but our simulations suggest that especially when sample size is an issue, more is not necessarily better. In combination with plots of raw phenotypic variance against the environment, the researcher can use informed judgement. A simple approach would be to take the total number of environments (*N_x_*) and divide it by a predetermined number, for example by 10, 7, 5, 3 or 1 (i.e. heterogeneous), or fitting a homogeneous residual variance. Information criteria can also be used to compare different means of grouping (e.g. equal‐interval groups vs. groups based on natural breaks in the data) or, if possible, to compare discretization versus a continuous change in residual variance. It should be borne in mind that the more discrete groups, the more degrees of freedom are used and the higher the risk of overfitting. Importantly, the chosen residual structure should be an informed one, and this should be communicated to the reader.
3. *Replace the environmental covariate in the RRM with environment‐specific mean phenotypes.* When the trait in question does not respond strongly to the environment, estimates of I × E and the power to detect it may be downwardly biased (Gienapp, 2018). There may, however, still be undetected I × E and even G × E in the population, which may have implications for the ability of the population to genetically respond to selection. The mean phenotype in a given environment can be used in certain contexts as a substitute for the “real” environmental driver and in that way serve as a “yardstick” for testing whether I × E and/or G × E exists in the population (Gienapp, 2018; Ramakers, Culina, Visser, et al., 2018; but see discussions in Brommer, 2019 and Ramakers, Culina, Visser, & Gienapp, 2019).
4. *Do a power analysis by simulation.* Whenever the RRM fails to pick up statistical evidence for I × E, the question arises whether this is due to a true lack of I × E or the lack of statistical power. Simulations can shed light on this. One can simulate a population with differing *N_x_*, *N_o_* and $\sigma_{b}^{2}$ and "play around" with parameter values to infer how likely one was to detect I × E in the real data in the first place.

## 5 CONCLUDING REMARKS

We provide a simulation‐informed set of guidelines that students of behavioural or life‐history plasticity may adopt to successfully estimate environment‐specific individual variances (I × E) and/or genetic variances (G × E) using random regression tools. When sample sizes are reasonably large, a simple information‐theoretic approach to selecting the best model should help one arrive at the best model explaining the data. We note, however, that when sample sizes are too small, even the most efficient model will not be able to estimate I × E reliably. Defining what is a decent sample size is beyond the scope of this study and has been elegantly demonstrated in previous studies (Martin et al., 2011; van de Pol, 2012). Nevertheless, we encourage researchers to always thoroughly document all statistical procedures (e.g. though R scripts) and report sample sizes, effect sizes and the precision of their estimates, which in the long run will serve the scientific field by enabling biological synthesis across study systems, for example in the form of meta‐analysis.

## AUTHOR CONTRIBUTION

J.J.C.R., M.E.V. and P.G. conceived the study. J.J.C.R. performed the analysis and wrote the manuscript. M.E.V. and P.G. critically reviewed and commented on the manuscript.

## Data Availability

The DOI for our data (Vlieland population) and R scripts is https://doi.org/10.5061/dryad.tqjq2bvts. Data for the Hoge Veluwe population were published previously (Ramakers, Gienapp, & Visser, 2018).
