# Supplementary material for: Quantifying individual variation in reaction norms: Mind the residual
Source: J Evol Biol. 2019 Dec 9;33(3):352–66. doi: 10.1111/jeb.13571 (PMC7079083; doi:10.1111/jeb.13571)

**Supplementary material**

Supplementary figures to the manuscript entitled “Quantifying individual variation in reaction norms: mind the residual”. *Journal of Evolutionary Biology.*

Jip J.C. Ramakers, Marcel E. Visser & Phillip Gienapp

***Figure S1****. Estimated slope variances (median + 95% CI; left-hand axis) and proportion of significant (p < 0.05) models (asterisks, right-hand axis) from different random-regression analyses on different simulated scenarios (N_o_ = 2 and N_x_ = 40 in all panels; see Table 6.1 main text). From top to bottom: the strength of the correlation between phenotypic variance (*$\sigma_{z}^{2}$*) and the environment (x) increases through changes in* $r_{\sigma_{e}^{2}, x}$ *(a–c: 0.01; d–f: 0.2; g–i: 0.5; j–l: 0.8); from left to right: simulated slope variance (*$\sigma_{b}^{2}$*) increases (a,d,g,j: 0.003; b,e,h,k: 0.3; c,f,j,l: 1.0), denoted with horizontal dotted lines. The horizontal axis displays the environmental variability (*$\sigma_{x}^{2}$*); different colours and symbols display the estimates from models with different residual structures (blue: homogeneous residual structure; grey and yellow: heterogeneous residual structure based on similar environments and through random grouping, respectively, using groups of 5 (circles) or 10 (triangles) environments).*


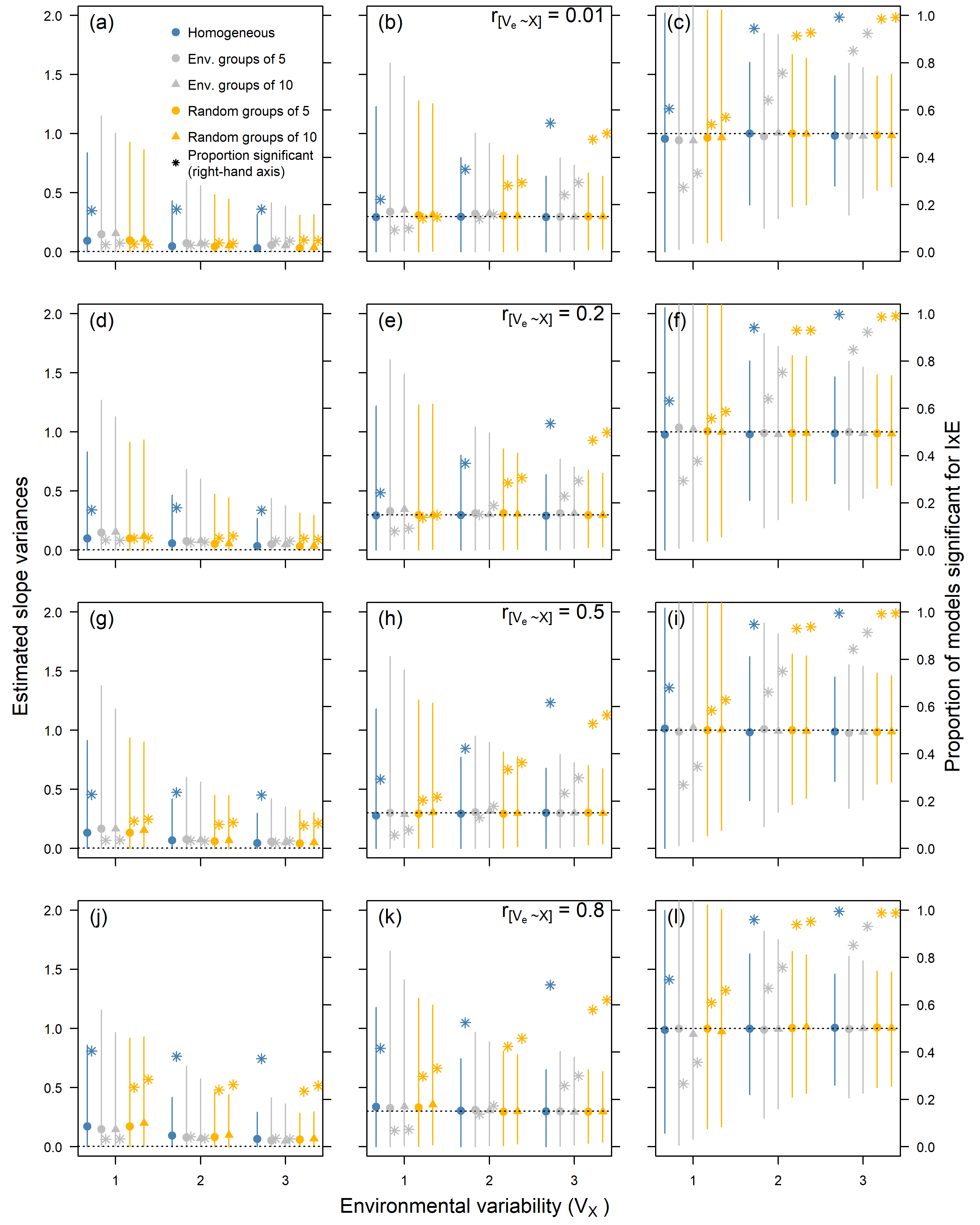


***Figure S2****. Estimated slope variances (median + 95% CI; left-hand axis) and proportion of significant (p < 0.05) models (asterisks, right-hand axis) from different random-regression analyses on different simulated scenarios (N_o_ = 5 and N_x_ = 40 in all panels; see Table 6.1, main text). From top to bottom: the strength of the correlation between phenotypic variance (*$\sigma_{z}^{2}$*) and the environment (x) increases through changes in* $r_{\sigma_{e}^{2}, x}$ *(a–c: 0.01; d–f: 0.2 g–i: 0.5; j–l: 0.8); from left to right: simulated slope variance (*$\sigma_{b}^{2}$*) increases (a,d,g,j: 0.003; b,e,h,k: 0.3; c,f,j,l: 1.0), denoted with horizontal dotted lines. The horizontal axis displays the environmental variability (*$\sigma_{x}^{2}$*); different colours and symbols display the estimates from models with different residual structures (blue: homogeneous residual structure; grey and yellow: heterogeneous residual structure based on similar environments and through random grouping, respectively, using groups of 5 (circles) or 10 (triangles) environments).*


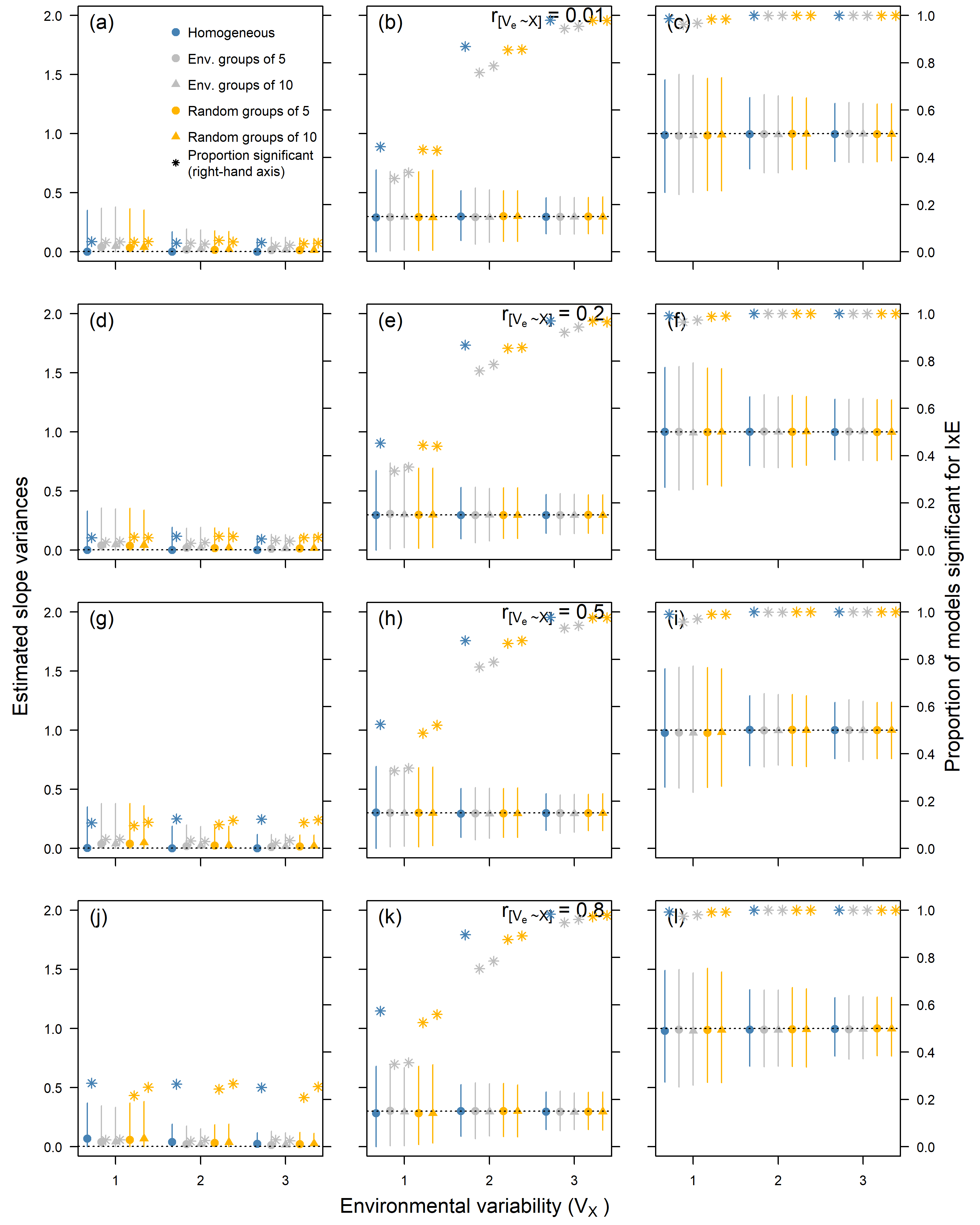


***Figure S3****. (a,b) Estimated reaction norms (posterior median estimates for each female) and (c,d) estimated phenotypic variance (combined permanent-environment and additive genetic effect; posterior medians and 95% HPDI) for great tit laying dates against individual-centred temperatures in HV (a,c) and VL (b,d). Estimates were obtained from Models 5 (HV) and 4 (VL) in Table 3 (main text). Temperature-specific variance estimates (i.e. in the j^th^ environment) were estimated as* $\hat{\sigma}_{z,j}^{2}=\hat{\sigma}_{a}^{2}+2\hat{\sigma}_{a,b}T_{j}+\hat{\sigma}_{b}^{2}T_{j}^{2}$*, with a and b representing intercepts and slopes, respectively, and T representing the (individual-centred) temperature.*


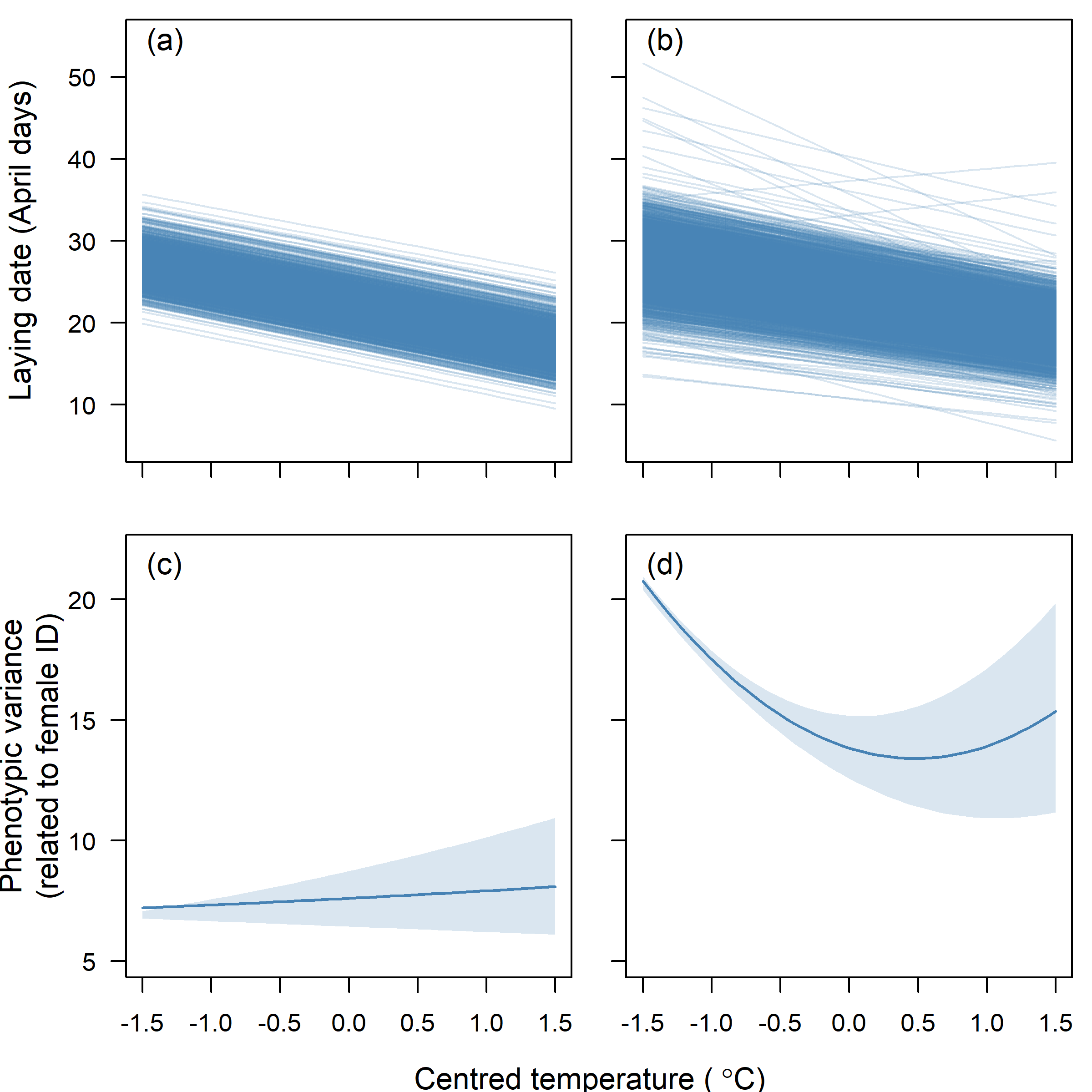

Supplement: Supplementary file 1 [file JEB-33-352-s001.docx]
